# Select Medico-Chirurgical Transactions; or a Collection of the Most Valuable Memoirs Read to the Medico-Chirurgical Societies of London and Edinburgh, &c. &c. &c.

**Published:** 1831

**Authors:** 


Art. VI.—*Select Medico-Chirurgical Transactions; or a Collection of the Most Valuable Memoirs read to the Medico-Chirurgical Societies of London and Edinburgh; the Association of Fellows and Licentiates of the King and Queen's College of Physicians in Ireland; the Royal Academy of Medicine of Paris; the Royal Societies of London and Edinburgh; the Royal Academy of Turin; the Medical and Anatomical Societies of Paris, &c. &c. &c.* —Edited by Isaac Hays, M. D.—Philadelphia: E. L. Carey and A. Hart.—pp. 420.

The volume which has been offered to the American public, under this title, is the first of a series of volumes contemplated to be published by the enterprising gentlemen whose names appear on the title page.

In this country, the physicians of every grade select the medical journals as the medium of offering their communications to the public. In Europe, the case is different. The transactions of learned societies, occupy a much higher rank, and are therefore generally selected by those whose talents or reputation can procure for their writing an insertion. The expensive character of these publications, places them beyond the reach of the profession in this country; and they are hence indebted for the information they convey, to the occasional and imperfect analyses of them, which appear in the medical journals.

This publication is an effort to supply this defect, to a certain extent, by giving a selection of the most valuable of the papers, read before the different learned societies of Europe.

The first article in this collection, is a paper by W. Lawrence, Surgeon to St. Bartholomew's Hospital, read before the Medico-Chirurgical Society of London—*Observations on the Nature and Treatment of Erysipelas, illustrated by cases.*

By Erysipelas, the author understands inflammation of the skin, either alone or in conjunction with that of the subjacent adipose and cellular tissue. Like other inflammations, it varies in extent, and its degrees are described under the familiar names of erythema, erysipelas, and phlegmonous erysipelas. The first, a simple eruption of the skin; the second, a more violent inflammation extending into the cellular membrane, and producing effusion and vesication; the last, involving both the cellular and adipose membrane, and causing suppuration and mortification of the former.

His description of simple erysipelas we shall pass over, except the following description of the appearances after death:

"In simple erysipelas the red colour disappears as soon as the circulation stops. The cuticle, if not already separated by vesication, soon loses its adhesion to the cutis, and the surface of the latter, in an advanced stage of the affection, has a livid appearance. The texture of the skin presenting a reddish or brownish tint, is loaded with serum, and softer than in the natural state. Serous effusion is found in the cellular tissue; and its vessels, as well as those of the skin, are distended. Sometimes we unexpectedly discover suppuration where the case has appeared during life, to be simple erysipelas, and no symptoms indicating formation of matter have been noticed."

These remarks are illustrated by the following account of the appearance of the diseased appearance, in a patient who died on the third day of an attack of the disease, supervening on a severe wound of the scalp:

On the 18th, erysipelas appeared in the arm and neighboring part of the forearm, the affected parts being tumid, bright red, and very painful. He died on the 21st, the erysipelas not having extended beyond the parts first affected. He had been bled three times in the arm. On examination after death, the veins were found perfectly healthy. The skin and adipous membrane of the inflamed part were pale and natural; a large effusion of yellow and somewhat thin, but good pus, was found in the cellular structure connecting the adipous membrane to the muscles along the whole back and inner part of the arm. The matter did not form a distinct collection, but two or three table spoonfuls were found in one part, and it was thence diffused through the cellular substance, without any definite boundary, and seemed to have gravitated into the place where it was found, in consequence of the position of the limb."

Phlegmonous erysipelas differs from the former in the higher degree, and deeper extent of the inflammation, and in the greater severity of the general symptoms. As the more aggravated forms of the disease are rarely to be met with in this country, we shall take the liberty of quoting his description of it;

"The redness is deeper than in simple erysipelas, often with a brownish or dark livid tint; the discolouration is often irregular, giving to the part a marbled appearance; the tumefaction is more considerable, the whole depth of the adipous and cellular textures being loaded with effusion, so that an arm or leg appears of twice the natural size; the sensation of heat and pain, at first slight, is aggravated to a very severe degree, and may be accompanied with throbbing. The swollen part at first yields slightly to the pressure of the finger, but subsequently becomes tense and firm. Vesications form on the surface, often minute and miliary, with purulent contents; (see Cases 27, 28, 29;) frequently however the skin does not vesitate. (See Cases 22, 23, 24, 26.) Suppuration and sloughing of the cellular membrane soon come on, the skin becoming livid and covered with phlyctænæ, (see Case 36,) and the febrile symptoms are aggravated. These changes are not attended with increased swelling, elevation, and pointing, as in phlegmon; on the contrary, there is rather a diminution of tension, a subsidence, and a feel of softness in the part. At first the cellular texture contains a whey-like or whitish serum, (Cases 22 and 26,) which I have sometimes seen in the eyelids, almost of milky whiteness. (See Case 21.) The fluid gradually becomes yellow and purulent, and we often find it presenting all the characters of good pus, and very thick. The serum is diffused through the cells at an early period, and a mixture of serum and pus often fills a considerable portion of the cellular texture without any distinct boundary. (See Case 36.) Frequently matter is deposited in small separate portions, forming a kind of little abscesses, which often run irregularly in the cellular texture. Such small collections are sometimes found where lividity or phlyctænæ have not preceded, and where no external changes nor any aggravation of other symptoms have announced suppuration. During this process of suppuration, the cellular texture turns grey, yellowish or tawny, (Case 28,) and sometimes appears like a dirty, spongy substance, filled with turbid fluid; (Cases 22 and 29;) then losing its vitality altogether, it is converted into more or less considerable fibrous shreds of various size and figure, which come away soaked with matter like a sponge. The integuments over a large slough of this kind, being deprived of their vascular supply, become livid, and often lose their vitality.* The suppurating and sloughing processes go on to a great extent when an entire limb is affected, sometimes completely detaching the skin, and often separating it through a large space; occasionally penetrating deeper, passing between the muscles, causing inflammation of them, suppuration between them, and often sloughing of the tendons. When the substance of a limb is thus generally inflamed, the joints situated in the affected part do not escape. Inflammation of the synovial membrane, effusion of matter into the joint, and ulceration of the cartilages take place.† An inflammation of such extent and violence cannot fail to excite the most serious sympathetic affections, among which may be mentioned disturbance of the nervous system, causing symptoms of typhoid character, inflammation of the lungs or pleura, of the intestinal mucous membrane, producing diarrhœa, or of the peritoneum, and inflammation or suppuration of other organs. The combination of the primary and secondary affections is speedily fatal.‡ If, however, the patient should recover after tedious suppurations and discharge of sloughs, the parts, which have been inflamed, are so changed in structure, and skin, fascia, muscles, tendons, and bones, are so unnaturally agglutinated and fixed, after the extensive destruction of the connect-

---

* Baron Dupuytren, who has particularly noticed this kind of secondary gangrene in his clinical lectures, explains the reason why it does not occur in the head. The seat of phlegmonous erysipelas, or, as he calls it, *diffused phlegmon*, in that situation, is the cellular texture between the fronto-occipital aponeurosis and the pericranium. The sloughing of that texture does not affect the nutrient vessels of the scalp, which lie exteriorly to it, immediately under and almost in the integument itself.

† In a case observed by Mr. C. Hutchinson, the cartilages of the femur and tibia were absorbed; at least there was a complete anchylosis of the knee.—Practical Observations, 2d edit. p. 115. Pus in the ankle-joint; the articular cartilages absorbed, and the tibia carious. Bibl. Med. Sept. 1827. p. 331. In another case, the elbow and shoulder-joints were inflamed, and the articular cartilages ulcerated. Ibid. p 333.

‡ The mortality under this affection has sometimes been truly alarming. Of fifteen cases which occurred in Plymouth Dock Yard, during August and September, 1824, twelve ended fatally. In the bodies of those who were examined after death, the following morbid appearances were found; viz: sero-purulent effusion into the thorax, with inflammation of the lungs; similar effusion into the abdomen, inflammation of the mucous membrane of the ilium and colon, and of the liver. In one case there was an abscess with a pint of pus round the kidney; and in another, the kidney was converted into a fluid like thick cream. The head does not seem to have been examined in any case.—Dr. Butter's Remarks on Irritative Fever, &c. Devonport, 1825. Sero-purulent effusion into the chest, inflammation of the lung, and of the mucous membrane of the ilium and colon, were the appearances observed in some fatal cases described by Messrs. Moulin and Guibert in the Nouvelle Bibl. Med. September, 1827.

ing cellular texture, that the motions of the part are permanently and seriously impaired.

The affected part which is at first firm and almost brawny to the feel, becomes softer when diffused suppuration and matter mixed with sloughs are under the skin."

As to the seat of the disease, Mr. Lawrence places it in the skin and the cellular membrane, admitting that the fascia may occasionally, although rarely, become involved in the disease, in opposition to the opinion of Messrs. Arnott, Earle, and Hutchinson, who place its chief seat in the fascia.

Mr. L. infers the nature of this disease to be inflammatory. It resembles inflammation in its four leading characters, redness, swelling, heat, and pain, and in its effects, effusion, suppuration, and sloughing. He is disposed to place it between the exanthemata and phlegmon. It is more circumscribed than the former, and more diffused than the latter. The exanthemata are confined to the skin, and phlegmon primarily attacks the cellular membrane, while erysipelas simultaneously attacks both. The most important distinction between erysipelas and phlegmon, appears to arise from the difference of the effusion, serum being poured out in the one case, and coagulating lymph in the other.

The author is not disposed to agree with those who regard erysipelas as a distinct species of inflammation, capable of attacking other structures besides the skin. The symptoms of erysipelas are so clearly referable to the peculiar structure of the skin, and cellular structure, that we certainly could not expect to meet with it in parts as differently organized, as the serous membranes and the viscera. There is some analogy between the structure of the mucous membranes and the skin, and we might consequently expect some similarity in their disases; but although these surfaces mutually exert a powerful influence over each other, we see nothing like a similarity in their diseases. An erysipelatous opthalmia has been described, but it bears very little resemblance to true erysipelas; and when the disease attacks the face in its most violent form, it seldom extends to the eyes or the mucous membrane of the mouth. It is indeed frequently accompanied by inflammation of the mucous membrane of the fauces, but this affection differs in many important particulars from the disease under consideration.

The author thinks there is a marked difference between phlegmonous erysipelas, and diffuse inflammation of the cellular texture, as described by Dr. Duncan. This affection sometimes appearing spontaneously, and sometimes conseqnent upon external injury, spreads deeply in the cellular texture, and among the muscles, causing extensive sloughing and suppuration; but in this disease, the skin is either unaffected, or affected only secondarily from the extension of the inflammation of the cellular membrane. In some of the cases, the skin was unaffected, although extensive suppuration of the cellular membrane, and in one case, mortification was ascertained by dissection. The muscles are also frequently affected, being changed in color, and sometimes so soft as to be easily lacerated.

Mr. L. looks upon erysipelas as decidedly an inflammatory disease.

"I am quite at a loss to discover in this affection those marks of debility which some have insisted on. Erysipelas, like any other inflammation, may occur in old and feeble persons, and the effects of the disease, when aggravated by injudicious treatment, or protracted from any cause, will soon weaken the most robust; but however weak the patient, the local disturbance is one of excitement; there is increased activity in the circulation of the part, clearly marked by all the symptoms. Indeed, speaking of the part, I am unable to recognize debility as the cause of any inflammation whatever, and in reference to the seat of disease, I regard the expressions of passive and asthenic inflammation and venous congestion, as either unmeaning, or calculated to convey erroneous notions."

He thinks phlegmon and erysipelas are not different in their essential nature, and we have a confirmation of this opinion, in the fact, that these diseases so frequently run into each other, that in many cases we scarcely know whether to refer the disease to one or the other. The diagnosis between the extremes is easily established, but between these, there is a numerous class of cases that may as well be referred to the one as the other. "Thus we occasionally find sloughs in abscesses, that must be considered phlegmonous; and we see circumscribed collections of matter in erysipelatous inflammations. Erysipelas is sometimes confined to one spot, while phlegmonous inflammation may extend its circumference, attacking fresh parts successively, so as to produce suppurations, and thus spread over a large part, or the whole of a limb;" and in chronic cases, the diagnosis is still more obscure.

In like manner, phlegmonous erysipelas is easily distinguished from cellular inflammation; but they insensibly run into each other; as this last does also into simple edema.

The causes of erysipelas are analogous to those of other inflammatory diseases; accidental or constitutional causes determining, whether the skin, or other parts, shall be the seat of the disease. In most cases of this disease, the digestive and biliary organs are much deranged, sometimes as the cause, and frequently as the consequence. The great majority of cases of phlegmonous erysipelas are idiopathic, produced by local irritation. Traumatic erysipelas may always be prevented by suitable dressings and diet. Simple erysipelas more frequently arises from internal causes, especially disorders of the primæ viœ or liver.

*Treatment.*—The author's opinion of the disease, would lead, of course, to an antiphlogistic mode of treatment, venesection, local bleeding, purging, low diet, with saline and diaphoretic medicine. Vigorous treatment at the commencement will frequently cut short the disease. In slight cases, the necessity of immediate relief is not so urgent, as in affections of the vital organs; yet the serious consequences which frequently result, render an efficient mode of treatment at all times advisable. When it proceeds from visceral affection, the removal of the cause will indeed cure the disease; but even in these cases, venesection is very frequently useful, both to the internal and cutaneous disease. The following remarks should be impressed on the mind of every young practitioner:

"It will appear from the foregoing remarks, that in contending for the inflammatory nature of erysipelas, and for the propriety of treating it antiphlogistically, I do not mean to recommend, that measures equally active, and, in particular, that bleeding, whether general or local, are to be employed in all cases. In young persons, in the robust and those of full habit, in instances where the pulse is full and strong, or when there is head-ache and white tongue, in erysipelas of the head, attended with symptoms denoting affection of the sensorium, and more especially in the very beginning of the affection, venesection will be proper, and it may be necessary to bleed largely, to repeat the evacuation, or to follow venesection by local abstraction of blood. (Cases 7, 8, 9, 11, 12, 13, 17.) Under such circumstances, the other parts of the antiphlogistic plan must be also employed, that is, the alimentary canal should be cleared by an active purgative, which may be followed by salines and antimonials, with the occasional use of milder aperients; and low diet should be enjoined. Nothing can be more different from such a case, than that of an elderly person with a small and feeble pulse, in the advanced stage of the disease. The interval between these extremes is filled by numerous gradations requiring corresponding modifications of treatment. The antiphlogistic plan itself embraces a wide range in point of degree; from blood letting, local and general, with purging, vomiting, the free use of mercury and antimony, and low diet, to the exhibition of a mild aperient, with some saline medicine. (Case 19.) The treatment of erysipelas, like that of any other inflammation, must be modified according to the age, constitution, previous health and habits of the patient, and the period of the complaint. In asserting generally that the antiphlogistic treatment is proper, I speak of the beginning of the disease, when the original and proper character of the affection is apparent; and I am decidedly of opinion that, in some shape or degree, such treatment will always be beneficial in that stage."

Of the different modes of local depletion, Mr. L. prefers cupping, where it can be applied or borne. The apprehension expressed by Willan and Thompson, respecting the bite of leeches, the author asserts is perfectly groundless.

In support of the practice of bleeding, the author cites the practice of many distinguished medical writers, and, among others, of the French physicians; who not only apply them to the inflamed surface, but, if there be febrile excitement, apply them also to the epigastrium. At the same time, he admits that an array of names, equally formidable, may be advanced on the other side, recommending a course diametrically opposite. Mr. L. can conceive of no satisfactory principle upon which these opposite opinions may be reconciled.

Mr. L. places little confidence in emetics, without the aid of more efficient measures, although he gives them when the tongue is furred, and fever is present, with sickness or nausea. Under similar circumstances, he gives calomel, either alone, or in combination with some powder, until the bowels are opened, and the tongue becomes clean.

When the inflammation is arrested, tonic remedies are to be resorted to, with caution. If injudiciously used, the fever is renewed, and much injury frequently produced. In some cases, they are useful at first, but soon cause a return of the complaint, if persevered in. Where a doubt exists of the propriety of this class of remedies, the carb. of ammonia may be given without danger, and with marked advantage—Next to this remedy, he prefers sulp. of quina, and occasionally admits, for a short time, of the sparing use of wine.

Local applications produce but temporary relief. Before the inflammation is developed, cold applications are useful, by lessening the heat of the skin; and, after it is established, warm fomentations, if steadily used for a long time, are very soothing. Of the use of blisters, he says but little, although he has tried them three or four times in simple erysipelas of the extremities, in the manner recommended by Dr. Physic, (although without recognizing the claims of that gentleman,) and with success. Compression of the limb by bandages, he condemns most unequivocally, as a practice fraught with the most dangerous consequences.

The treatment of phlegmonous erysipelas corresponds with that above laid down, except that local bleeding is much preferable to the lancet. Venesection, Mr. L. remarks, exercises but little influence over cellular inflammation; and, therefore, when he resorts to it in this disease, it is rather with a view to the general febrile symptoms, than to the local disorder: for, if the disease is not speedily arrested, further depletion is of no avail, and the disease will spread in spite of bleeding, whether local or general.

The treatment, however, upon which the author relies, more than upon any other, is, by incisions through the inflamed skin and subjacent adipose, and cellular membrane.

"These incisions are followed very quickly, and sometimes almost instantaneously, by relief and cessation of the pain and tension; (Cases 24, 28, 30, 31;) and this alleviation of the local suffering is accompanied by a corresponding interruption of the inflammation, whether it be in the stage of effusion, (Cases 21, 22, 23, 24, 26, 28, 30, 31) or in the more advanced period of suppuration and sloughing. (Cases 20, 22, 25, 27, 29.) The redness of the skin is visibly diminished during the flow of blood from the incisions; in twenty-four hours it has usually disappeared, and the skin itself is found wrinkled from the diminution of the general inflammatory tension. The immediate relief, although very desirable to the patient, is however of less consequence than the decided influence of the practice in preventing the further progress of the disorder; and this important result has never failed to occur, within my experience, when the case has been a proper one for the practice, and the state of the patient has admitted of its being fairly tried. The cases already referred to furnish the clearest evidence on these points.

The treatment by incision is suited to various stages of the complaint; but it is employed to great advantage at the beginning, since it prevents the further extension of inflammation, and the occurrence of suppuration and sloughing. (Cases 30, 31, 24, 26, 28.) The redness and swelling gradually subside; the surface of the cut granulates, and it heals rapidly. At a more advanced period, the incisions limit the extent of suppuration and gangrene; and at a still later time, they afford the readiest outlet for matter and sloughs, and facilitate the commencement and progress of granulation and cicatrization. When the matter has been fully discharged, and the sloughs, whether of the skin or cellular membrane, have separated, a healthy granulating surface is left, and no great difficulty is experienced in effecting cicatrization, unless the destruction of the skin should have been very extensive, when the cicatrix forms slowly, and is liable to give way again."

The author recommends incisions only in cases of phlegmonous erysipelas. The milder cases, and the early stages of more severe ones may be trusted to antiphlogistic remedies; but, as the disease is often insidious, we should be on our guard and not suffer it to proceed too far, before the remedy is resorted to.

In erysipelas of the face, this mode of treatment is not necessary, unless the disease attack the cellular membrane immediately above the eyebrows. In this part, it is frequently followed by suppuration, sloughing, and mortification of the skin, and here this mode of treatment is frequently necessary. In erysipelas of the scalp, when the disease attacks the cellular membrane under the aponeurosis, this mode of treatment is imperiously called for at an early period.

After the incisions, the parts should be covered with warm fomentation cloths, until the bleeding ceases; a poultice should then be applied, until suppuration is established; and, if the discharge does not soon take place, lint, spread with some stimulating ointment, should be applied under the poultice, until that event takes place. When suppuration is established, large portions of cellular membrane are often discharged, and extension of the incision is frequently necessary to facilitate their separation. When this process is completed, especially if the sloughing is extensive, the pressure of a bandage will be serviceable in promoting the cure.

This mode of treatment has been in some cases successfully extended to the erythema anatomicum. In one case of this kind, under the care of Mr. Earle, incisions were made on the sixth day, when the case of the patient was perfectly desperate. The limb was greatly swollen, and extremely painful from the arm to the shoulder, the pulse rapid and small, and the countenance anxious and haggard. As a last resource, three incisions were made, one in the arm, and two in the fore arm, which bled so copiously that three pounds and a half of blood were estimated to be lost; when so sudden and complete was the relief, that in six hours he was considered perfectly out of danger.

The mode of making the incisions, adopted by Mr. Lawrence, is somewhat different from that pursued by Mr. Hutchinson. The latter recommends a number of smaller incisions, under the impression that the relief, in severe cases, extends to but a small distance from the incision; while the former thinks that one incision, carried through the whole length of the inflamed part, will answer every purpose, and will be less objected to by the patient. Mr. H. remarks that there will be less danger from hæmorrhage, if the incisions are of smaller extent; and, if extensive sloughing or suppuration is threatened, or has already taken place, the disease will be more readily relieved. The suggestion, respecting hæmorrhage, is certainly of consequence; for, when the inflammation is considerable, such is the tendency to hæmorrhage, that, in some of the cases detailed by Mr. L., the flow of blood was very alarming, and, in one case, was probably fatal.

Dr. Dobson, surgeon to the Royal Hospital, Greenwich, has adopte a modification of this mode of treatment, consisting of numerous punctures in the inflamed part. Punctures, varying from ten to fifty in number, are carried, from two to four tenths of an inch, into the cellular membrane; and this process is repeated two, three, or four times a day, according to the urgency of the case. A further account is given of this practice, in a subsequent part of this Journal.

The fourth article is a paper by Mr. John C. Ferguson—*Auscultation the only unequivocal evidence of Pregnancy;* and the fifth is a paper from the Dublin Hospital reports—*Observations on the Utero-Placental Circulation, and the phenomenon of Placental Soufflet*—By Evory Kennedy, M. D. On applying the ear, either immediately or by the interposition of the stethoscope, to the abdomen of a pregnant woman, there will be heard, at some part of the uterine tumour, provided the pregnancy be sufficiently advanced, a peculiar sound, bearing more or less resemblance to the action of a bellows, and denominated, by its discoverer, Kergaradec, the *Placental Soufflet.* The following account of this phenomenon is given by Dr. Kennedy:

"Although the placental soufflet does not always exhibit the same unvarying characters, yet these are sufficiently marked to render it recognizable in almost every case. It assumes all those varieties described by Lænnec under the head of Bellows-sound. The bellows-sound, properly so called, likened by this author, to the continuous murmur similar to that of the sea, familiarly exemplified by the application of a large shell to the ear; the rasping or sawing sound, which is occasionally found so exactly imitated as to lead the listener to imagine that an artisan is at work quite close to him; and the musical or hissing sound, partaking of the vibrating character so well described by the above named author. In addition to these, I have at times observed this phenomenon resembling the cooing of a dove, but this is particularly rare. A more frequent peculiarity which I have noticed, is a strange drone, resembling that of a bagpipe accompanying the sound, coexisting without interfering with it. The most constant form we meet with, however, is a combination of the bellows, or the sawing with the hissing sound, commencing with one of the former, and terminating with the latter, which is in some cases so lengthy and protracted, that the last soufflet is audible when the subsequent one commences, seeming thus to run into each other. These sounds are, from the distension of the uterus and consequent facility of examination, easily discovered in advanced pregnancy; and although not so loud and sonorous in the earlier stages, yet to the practiced ear are quite as distinct. I have not observed any of the above mentioned sounds peculiar to particular stages of pregnancy, having detected them indifferently throughout all. This phenomenon is perceptible over an extent of surface greater or less, according to circumstances; it may have its seat in any part of the uterus, and cases will occur, although if proper precautions be had recourse to, very rarely, where we shall not be able to detect it."

Dr. K. supposes this sound to be produced by the passage of the blood through the arteries of the uterus, to which the placenta is attached, and through those of the maternal part of the placenta. It will be recollected by the medical reader, that the circulation in the fœtal and maternal part of the placenta are independent of each other, each being kept up by the action of the heart of that system to which it belongs; and that the action of either may be kept up for some time, although that of the other be suspended or destroyed. It is the latter of these that the author supposes to give rise to the placental soufflet, and his opinion is sustained by the following facts.

1. This sound is heard only when the placental circulation exists, and ceases when the vessels which connect the uterus and placenta cease to transmit blood; as when the uterus is empty and contracted, or when the circulation ceases, in consequence of the death of the fœtus.

2. The sound is always heard in that part of the uterus to which the placenta is or has been attached.

3. The sound is synchronous with the mother's arterial pulsation.

4. The sound does not necessarily terminate with the life of the fœtus, nor with the expulsion of the placenta, but frequently continues after those events, as long as the contraction of the uterus remains imperfect. The soufflet, also, is very frequently observed in after pains, sometimes as late as two days after delivery. Dr. K. remarks that it sometimes continues for a considerable time after the vitality of the fœtus is destroyed, as it is perceptible after the action of the fœtal heart has ceased, and has continued to be so, when the marks of putrescency on the children proved them to have been long dead. From this fact, accompanied with the circumstance that the placenta, under these circumstances, is frequently discharged, loaded with fluid blood, he infers that a connection between the uterus and the placenta, sufficient to preserve the vitality of the latter, is frequently kept up after the fœtal circulation has entirely ceased.

But, although the sound continues after the interruption of the fœtal circulation, it is very materially altered in its character—"abrupt, of short continuance, and wanting the lengthened terminating whiz, of the perfect placental soufflet."

Persons unaccustomed to the use of the stethoscope should bear in mind that they may be deceived by other sounds, either from their resemblance to the placental soufflet, or the effect they have in concealing it. The *respiratory murmur* is sometimes conducted from the lungs, from the thoracic along the abdominal parietes. The *sonorous rale* somewhat resembles the *placental soufflet*, and this is conducted over the abdomen in the same way with the respiratory murmur. We may distinguish these by their not being synchronous with the pulse; and by tracing the sound along the abdomen, it becomes more distinct, as we approach the chest.

The abdominal aorta, and its large branches, sometimes give a sound more nearly resembling the *bruit de soufflet*, although still very easily distinguished by one accustomed to the sound. This sound is rarely heard in the aorta; and when it occurs in the iliac arteries, it is generally heard on both sides. The *placental soufflet*, also, may be heard over a larger portion of the abdomen, and is not confined to the immediate vicinity of the artery.

The *placental soufflet* and the fœtal heart are seldom heard before the fifth month of pregnancy, although, by accurate examination, they may be distinguished as early as the tenth or twelfth week. The former is generally heard in one or other of the iliac regions, though it may occur in any part of the abdomen; but, wherever it does occur, for obvious reasons, it remains stationary during the whole of pregnancy. The fœtal heart, on the other hand, is constantly changing its position.

The importance of these sounds, as a test of pregnancy, will be obvious to every one. It is now acknowledged, by all writers on the subject, that there is no unequivocal test of this condition; but, wherever these two sounds can be heard, there can be no longer a doubt, and they can almost always be ascertained, where pregnancy is much advanced; the stethoscope therefore, may be looked upon as a test of universal application.

In protracted labors, where the life of the mother is in danger, this instrument may be satisfactorily applied, to ascertain the life of the child, and thus to learn whether any objection exists to expediting the labor, by destroying the infant.

Dr. K. further remarks, that in the Cesarean section, the *soufflet* may act as a guide, and thus enable us to avoid wounding the large vessels; a circumstance which, in the case recorded by Dr. Henderson, produced death, by hæmorrhage.

Passing over several papers, we come to one *on the comparative infrequency of Urinary Calculi among seafaring people*—By A. Copland Hutchison, Esq.

The following extracts will show how astonishingly small is the number of seamen affected by this disease. The average number of men, annually voted by parliament to man the British navy, from January, 1800, to December, 1815, was 132,000. The author, taking into consideration the number of men who were lost to the service, in consequence of sickness, wounds, and desertion, thinks that the whole number, at the disposal of this branch of the service, during this period, could not be less than 162,000.

"I have taken considerable pains to ascertain the prevalence of calculous disorders in the naval service, and the result of my inquiry is, that out of the mass of individuals of which it is composed, only *eight* cases have occurred in the period of the *sixteen years* before specified, all of whom had been operated upon in the naval hospital of Halsar, Plymouth, or Deal, and of whom one only died. In the other royal hospitals, namely, Yarmouth and Peignton, no patient laboring under Urinary Calculus having been admitted, I have purposely omitted taking them into the account, as well as the foreign hospitals of Halifax, Jamaica, Antigua, Barbadoes, Gibraltar, Malta, the Cape, and Madras; being satisfied, after the strictest examination, that the operation of lithotomy had never been performed at any of these establishments. If cases of this kind had occurred abroad, and owing to the unfavorable nature of the climate or other causes, the surgeons had deemed it advisable not to operate; the patient so circumstanced would have been forwarded to the great naval hospitals at home; and therefore, we shall be fully warranted in concluding, that eight cases of stone only had really occurred among the vast mass of seamen and marines composing our naval force at home and on foreign stations, during that eventful era, that is, between January, 1800, and December, 1815."

Mr. H. ascertained, from a reference to the books of these hospitals, that the number of seamen received into them, amounted to 96,000. Deducting, in round numbers, 10,000, for gunshot wounds and other accidents to which sailors are exposed in time of war, above the average of other hospitals, and we have only one calculous patient, out of nearly 11,000. A reference to the number of calculous patients, in other hospitals, will show how very small is the proportion of persons affected with these diseases, among mariners, and even this small ratio, it will be found, is very much diminished by investigating the history of the patients. It is, also, to be remembered, that the patients in these hospitals are all males; among whom the proportion of calculous patients is seventeen times greater than among females.

"Dr. Marcet states the proportion of stone cases received into the different British and Continental public hospitals, Norwich alone excepted, to be *one* for every *three* or *four hundred* patients of all descriptions admitted. In the Norwich hospital it appears to be *one* in every *thirty-eight* cases, a proportion prodigiously great, and which places in a striking point of view the untoward prevalence of this afflicting malady in the Norfolk district, over that of every other throughout Europe, as far, at least, as our present knowledge extends, from whatever cause that extraordinary circumstance may arise. The paucity of stone cases occurring in tropical climates has also been remarked by Dr. Marcet; and far as the fact already stated goes, of no patients labouring under this disease in question, having been admitted into our foreign hospitals, it tends to strengthen the observation.

Of the eight cases of calculous concretions before mentioned as having occurred out of the vast mass of patients admitted into our naval medical establishments in England, two were boys about 14 years of age, who had laboured under symptoms of stone for some years previously to their admission into the service, and into which they had recently entered, expressly for the purpose of deriving benefit from our magnificent institutions; one was a marine, about twenty-two years of age, who had been at sea a few months only; three were adult seamen, and the seventh a marine; but their length of service afloat *could not be at all* ascertained: the eighth and last case was a warrant officer, advanced in years, who had been serving in ordinary, that is, in a ship in harbour, for a considerable time previously to the operation, and the only case which terminated fatally.

In the admissions into naval hospitals, both officers, under the rank of captain, and privates are included, and excepting the case of one warrant officer, whose diet differs not materially from that of the seamen, it will be found that no officer has undergone the operation of lithotomy in any of these establishments. It is but just, however, to notice the cases of two naval gentlemen, the one a captain, the other an hospital surgeon, who were operated upon in London, as I have been since informed; but whether the first contracted symptoms of the complaint at sea or while residing on shore, I have not been fortunate enough to discover."

The following quotation will give the author's ideas of the cause of this remarkable exemption:

"On the ship's arrival in port, the men are amply supplied with good fresh beef, vegetables, and sound table beer, while vitualling and watering, which, however, in time of war is generally limited to a very short period, especially when commanded by an active and zealous captain. On these occasions, it is incredible to see what quantities of salt the seamen will use with their fresh beef. During their stay in port, and for some days after, each man is allowed a gallon of good beer; and at other times, when this wholesome beverage can no longer be procured, a pint of wine or half a pint of spirits in lieu thereof; the latter previously diluted with three portions of water, is served out daily to each man at two distinct periods.

The beef or pork commonly issued to the ship's company at sea is so highly salted, and frequently kept so long in its briny pickle, that its bland and nutritious juices are in a great measure exhausted. Excepting in ships of war of the first and second rate, a portion of one deck only is appropriated to the whole ship's company to sleep in, and this is consequently so crowded with hammocks, and the men so impacted together, (fourteen inches in width being the total space allowed to each individual,) that some dexterity is requisite to obtain ingress and egress to and from their beds. The lower deck being always the part allotted for repose, the ports are for the safety of the ship necessarily closed all night, and the temperature of the surrounding air is thereby so exalted, that the place becomes a kind of steam bath from animal exhalation, the men being literally immersed in their own perspiration.

Dr. Dobson remarks, that calculous disorders are much more frequently met with in the cider counties than in other parts of England; and as it would appear from what has been here advanced, that seamen who have rarely opportunities of indulging in the use of malt liquors, are in great measure exempt from urinary concretions, it may therefore be asked, whether all kinds of fermented liquors be not favorable to the production and accretions of such disorders?

From Dr. Marcet's and Dr. Prout's remarks, it would appear, that an active and healthy state of the digestive organs is one of the most effectual preventives against the formation of calculi; may it not therefore happen, in the instance of seafaring men, that the peculiarities of their regimen, and especially the great quantities of muriate of soda they habitually take with their food, contribute to produce this effect? or in other words, shall we be justified in imputing to the stimulus communicated to and maintained in the whole chylopoetic viscera by the muriate of soda, a power to counteract the aggregation of calcalous matter in the urinary organs, independently of any direct chemical agency?

It has been already stated, that seamen belonging to ships of war, are so closely impacted whilst in their hammocks, that they are continually suffused with perspiration during the whole period allotted to repose: and there is also such a perpetual mutation in the various stations appointed to the ships of war to cruize in, that few seamen escape their performance of their round of duty in the tropical and other hot climates, bearing an equal proportion to the time spent in the more temperate climates of Europe; and consequently there must be a much more profuse discharge from the exhalants on the surface of this class of men, than of those residing in Great Britain or in more northern latitudes.

I am the more particularly induced to notice these circumstances, because it has been ingeniously suggested among other causes, 'whether there may not be some essential connexions between the state of the cutaneous functions and the greater or less prevalence of this class of disorders?' "

"The life of a seaman is one of great activity, and often of considerable labour and exertion. I have frequently observed, in common with other officers, that sailors never fail to empty the bladder on the first symptoms of distention; and the facilities afforded them, as far as regards unmixed society and locality, favour greatly this salutary habit. It is also of importance to notice, that no description of people are less subject to dispepsia, or more prone to strictures in the urethra.

From various parts of the preceding premises, then, we may with some degree of probability infer, that animal food, combined with a certain portion of the muriate of soda, in conjunction with farinaceous aliment, on which seamen principally subsist, are favourably to the prevention of calculous aggregation.

To acquire this prophylactic property, it may be essential that the animal food should be saturated with salt previously to its use, as we learn from Dr. Wollaston, that when free from saline matter, animal food favours the generation of lithic acid, at least in carniverous birds; whether similar effects follow its application to the human stomach, has not yet been ascertained, I believe; but reasoning from analogy, we might be induced to conclude that such would be the consequence.

With respect to the practical inferences to be deduced from almost the total absence of calculous disorders in tropical regions, the exhibition of sudorifics would appear to be indicated, as offering a prospect of preventing the malady altogether, or of alleviating its further progress when once established. It is well known that the cuticular exudations are vicarious with the renal secretions, and the most superficial observer must have witnessed, that when the cutaneous discharges are abundant or increased, micturation is proportionably diminished. Dr. Wilson has remarked, that Dover's powder and tartarized antimony (which are powerful sudorifics,) when administered to individuals, invariably lessen the quantity of lithic acid in the urine.

From the foregoing observations it would appear, that exercise is not only conducive to general health, but acts as a preventive to the disease in question, and probably may be used with material advantage even when calculi are known to exist, the *quantum* of course to be regulated by the magnitude or irritation produced by the calculus on motion."

In addition to the information obtained at these hospitals, Mr. H. made inquiry of E. Home, Mr. Cline, and Astley Cooper. The two first had never operated upon a seafaring person, and the last had been consulted by one naval officer, laboring under evident symptoms of the stone, but whom the author afterwards learned had not been employed at sea for the last twenty years of his life.

Mr. H. mentions, that in the Royal Hospital at Greenwich, appropriated to decayed seamen, and containing 2,700 patients, the physician to the institution did not recollect of a case having occurred, during his professional attendance, for twenty-seven years.

Since that time, Mr. Hutchison has extended his inquiries to most of the seaport towns in England and Scotland, and ascertained, that in them all, there was scarcely an instance of the operation having having been performed upon a seafaring person.

*The Clinical Reports, of the Meath Hospital,* by Drs. Graves & Stokes, of Dublin, are exceedingly valuable; but we have room for but few extracts. As the plan of treating pneumonia, by large doses of tartar emetic, is almost unknown in this country, we will present to our readers, the remarks of these gentlemen upon this subject:

"The plan of treatment which we have found of the greatest efficacy in combatting simple pneumonia, is the use of the lancet, and the exhibition of the tartar emetic in full doses. As this latter forms a mode of treatment not yet extensively adopted in these countries, we shall put our experience of it on record.

The cases in which this treatment is most applicable, are those where the disease is in the early stages, where it occurs in strong constitutions, and lastly, where there is absence of gastric symptoms. This is a point of great importance, and we shall refer to it presently.

It is during the existence of the first stage of the pneumonia, while the crepitating rale is heard most distinctly, and before the affected portion of the lung sounds dull on percussion, that we find the remedy to answer best: six grains are generally administered the first day, and the dose is increased by two or three grains daily, until fifteen grains are exhibited in the twenty-four hours. Beyond this dose we have never found it necessary to go; but we have been able to persist in the exhibition of the remedy at this rate for many days, and with the best effects.

The cases in which we have found it necessary to continue this treatment longest, are those in which an acute pneumonia has supervened on a chronic catarrh. In one case of this kind, in which the pneumonia was double, one hundred and eighty grains of the tartar emetic were exhibited at the rate of twelve grains daily. In this case the tolerance of the remedy was completely established after the second or third day. Indeed, towards the termination of the disease, the patient's appetite was excellent, although he was taking the remedy at the rate mentioned. This we have often seen in other cases, a fact already observed by Lænnec.

We have very seldom observed abdominal irritation to follow the exhibition of the remedy, even where large quantities have been taken. In a few cases, after the subsidence of the pulmonary disease, colicky pains occurred, but these almost constantly yielded to stuping, mild laxatives, and opiates. In one case, however, they were so severe as to require blood-letting: the blood was cupped and buffed, and the patient recovered perfectly.

With respect to blood-letting in pneumonia, we rely more on it as a means of combating the inflammation than Lænnec appears to have done. Except in cases of pneumonia combined with hypertrophy of the heart, he considered bleeding more as a mode of preparing the patient for the exhibition of tartar emetic, than as calculated directly to remove the disease. We consider, on the contrary, general and local bleeding to be of primary importance, while the tartar emetic is a very useful adjuvant. Thus, on any sudden exacerbation of the disease, we do not trust to increasing the dose of the remedy, but have at once recourse to general or local bleeding, as the case may be; and we may here remark, that in the treatment of acute bronchitis and pneumonia, when occurring in the adult, local bleeding has been too little praticed in this country.

In some cases we find that the first dose of the remedy makes the patient vomit freely, but after a few more doses the medicine is borne well. But in the greatest number of cases a state of slight nauseau, without vomiting, is kept up, and continues for several days, and indeed as long as the remedy is administered.

Sometimes we have found both vomiting and purging to follow at first, but to subside after twenty-four hours. Diaphoresis is a rare effect, and we have often witnessed cases where the patient was taking from ten to twelve grains of the medicine daily, without vomiting, purging, or sweating; so that no effect could be observed, except a gradual reduction of the symptoms, and stethoscopic phenomena.

We generally find, in cases of simple pneumonia, when the disease is confined to the lower portion of one lung, that when we commence with the exhibition of six grains in the day, and increase this at the rate of a grain daily, we are able either to omit the remedy, or begin to diminish it in the course of four or five days. We seldom omit it suddenly, as more than once a severe relapse has followed this practice."

It is remarked, that in many cases of pneumonia, with great dyspnea and lividity, great improvement followed venesection and the exhibition of tartar emetic; but on the following day, no improvement in the condition of the lung was discovered by the stethoscope. These were all cases of partial pneumonia, with general bronchitis, and the improvement was owing to the beneficial influence of the remedies on the latter disease, while the patient was still in considerable danger from the former—a fact, of which every practitioner should be aware, in the treatment of pneumonic inflammation.

The last paper is one by Professor Marsh, of Dublin—*Observations on a peculiar convulsive disease, affecting young children, which may be termed Spasm of the Glottis.* Dr. Clark, in his commentary on the diseases of children, has described a spasmodic affection, which he terms "a peculiar convulsion in infant children." This disease, says the author, appears to consist primarily in spasm, occurring suddenly, and affecting the muscles of the glottis, as it increases in severity, extending to other muscles, particularly those of the fingers and toes; and, if neglected, or maltreated, is liable to lead to severe and universal convulsions. This disease, although alluded to by several authors, is not generally recognised, and the intention of the author is to direct the profession to it. The character and varieties of the disease are illustrated by cases, of some of which we proceed to give a concise account.

The disease sometimes exists alone, without any discoverable irritation or disease coexisting with it; more frequently however, it is complicated with painful dentition; with derangement of the digestive functions; with a cachetic state of the system, produced by bad diet or impure air; with fever, and occasionally, with effusion into the ventricles of the brain.

*Case* 1. "A child, eleven months old, remarkably healthy, and in appearance well thriven, had been for some time affected in the following manner: it had been observed, now and again, to awake suddenly from sleep in a state of alarm and agitation, to struggle for breath, and, after repeated efforts to recover from the paroxysm, with a long and sonorous inspiration; the convulsive effort was described to be severe, and the face to become swollen and purplish: these attacks occurred, at first, only on awaking from sleep, afterwards more frequently; sometimes without any perceptible cause, at other times, and more frequently, when she was vexed or was about to cry. On examining the child accurately, I could discover no deviation from a state of perfect health; the pulse and respiration were natural, skin cool and soft, tongue clean, the breath pure, the appetite good, the bowels regular; the alvine excretions exhibited the appearances usual in healthy children on the breast. She was playful and lively, but was startled unusually by any sudden noise; the four incisor teeth had appeared; the gums were neither swollen nor tender.

The treatment consisted in half grain doses of sulphate of quinine every sixth hour, and free exposure to the open air; the attacks became less frequent, and after some days ceased altogether. She has since (during a period of two years) remained in very good health.

*Case* 2. A patient whom I had been attending, happened to mention to me accidentally, that her child, about fourteen months old, was in the habit of awakening suddenly from sleep as if alarmed, breathed with difficulty, and made a loud noise like that in a fit of the hooping cough; she said also, that this occurred occasionally during the day, when the child was cross; she spoke of this lightly, as of an unimportant thing. I requested to see the child; it looked pale and unhealthy; the integuments were soft and flabby; the tongue coated; the bowels relaxed or confined; the stools green and curdy; the child was irritable and nervous, and seemed to suffer from dentition; the extremities were slightly swollen, and the thumbs placed firmly across the palms of the hands. I stated that I thought the child very ill, and that probably convulsions would take place.

Next day I was summoned in haste, and found the child just recovered from a severe paroxysm of general convulsions.

The gums over the projecting teeth were divided; leeches were applied to the temples, cold lotions to the head, and warm f mentations to the extremities; aperient medicines were given; the nurse, whose milk obviously disagreed with the child, was changed; and it was removed from the city to a healthy situation in the country; after one other attack of convulsions, recovery was speedy and complete.

In this case the disease was complicated with derangement of the digestive functions, aud also with painful dentition: the convulsive paroxysms, at first partial, increased in severity, and at length became general. The remedies which appeared to be ultimately and permanently efficacious, were, change of air and change of nurse."

*Case* 3. The advice of Dr. M. was requested for a fine child, 12 months old, which had been subject for four months to what was called "croupy breathing." The attacks, which occurred at first rarely, and only at night, produced so little derangement of the health, that very little attention was paid to them. But having increased in severity, medical aid was called in, when under the supposition of pneumonic inflammation, the patient was treated by bleeding, emetics, mercurial cathartics, and confinement to a warm room. When Dr. M. first saw the patient, the attacks were excited by the slightest cause, such as sudden noises, the removal of the child from the nurse's arms, &c. The struggle in breathing was protracted, the face became livid, and frequently this terminated by protracted and severe convulsions; the extremities were swelled and puffy, with a spastic rigidity of the fingers and toes: the digestive functions were very much deranged; and after one of the attacks strabismus had occurred.

The child, which had been weaned, was again provided with a wet nurse; the temperature of its room was lowered; and it was gradually accustomed to the external air. Under this treatment he rapidly recovered his health.

*Case* 4. A weak and delicate child, whose constitution had been very much impaired by previous disease, and the treatment to which he had been subjected, was subject to sudden attacks of dispnœa, in which the respiration was short and frequent, the head drawn back, and the sense of suffocation urgent. These attacks, which were protracted and severe, ended in a loud whoop or crow. When Dr. M. saw this child, he was confined to the bed with remittent fever; there was also spastic contraction of the flexor muscles of the fingers, the extremities were swollen and livid, and the child suffered severely from dentition. The gums were lanced, and under a treatment adapted to his febrile condition, the spasms subsided, and the child remained free from them for some weeks.

After some time, however, they returned with so much violence, that respiration was sometimes entirely suspended. Under these circumstances, it was determined to bleed the child until a sensible effect was produced upon the system. In consequence, there was no return of the spasm for ten days; but the system was prostrated, and at the expiration of this time, all the symptoms returned, accompanied with great debility. A tobacco enema at this time suspended the spasms, and there was no return of convulsive movement for a month. About this period, on a slight recurrence of the symptoms, the child was removed to the country, where he rapidly recovered.

In several cases which terminated fatally, there were scarcely any appearances found, on dissection, to account for the fatal event, the spasm of the glottis having subsided after death.

The author sums up his view of the disease in the following words:

"It seems to me not important to remark, that all the cases of this disease, which I have witnessed, have occurred in children either themselves exhibiting marks of the strumous diathesis, or sprung from scrofulous parents. This bears practically upon the subject, inasmuch as it enhances the value, in treatment, of pure air, healthy nutriment, and tonic remedies.

If we take a survey of the several cases of this disease, which have been stated, we learn that it varies much in degree, and that its complications are numerous. In its mildest and least complicated form, the spasmodic action is confined to the muscles of the glottis, and the treatment consists in improving the general health, and in giving tone to the nervous system. The symptoms in such cases will rarely fail to yield to some of the vegetable or mineral tonics, pure and bracing air, and a well regulated diet: in some cases, I have perceived, I

think, advantage to arise from some of the antispasmodic medicines, and amongst these, none has appeared to me more beneficial than the old-fashioned medicine, the Tinctura Fuliginis; but when the disease is complicated with painful dentition, derangement of the bowels, or any febrile movement in the system, the primary object of the treatment must be, to remove these accompanying ailments; until this be effected the treatment applicable to the spasmodic affection, though it may mitigate its severity, will fail to eradicate the disease. When the spasmodic symptoms extend themselves, and implicate the muscles of the extremities, the disease assumes a more formidable aspect, and soon, if not checked in its progress, paroxysms of general convulsions will establish themselves; in this stage the membranes of the brain become so frequently engaged, that the utmost vigilance on the part of the practitioner is required to prevent the occurrence of such mischief; yet it must not be lost sight of that the disease, even in its mildest form, is attended with danger: one case is recorded of sudden death during the spasmodic closure of the glottis; this occurred in a child who was otherwise in perfect health, and I have heard of several instances of the same kind. In every stage of the disease, therefore, we should be aware of and guard against the liability to sudden death; all needless sources of irritation should be avoided, and the child closely watched, and carefully held and supported during the paroxysm of dyspnœa. In irritable and passionate children the danger is increased. Dr. Johnson has stated to me, that he has seen a child, in a state of asphyxia caused by this disease, recovered from apparent death by the instantaneous application of artificial respiration."

In regard to the general design of the "Medico-Chirurgical Transactions," we think that the work may be made a very useful and interesting one. The wide field of selection which the nature of the enterprize affords, will secure to the editor every thing that can be expected from talents, experience, and observation. Every thing, therefore, depends upon his research and discrimination. Upon the general character of the selections, it is not easy to pass judgment, until we see the future numbers. A series of volumes of this kind should be made to give a complete view of the present state of medical science.

